# Facility type and surgical specialty are associated with suboptimal surgical antimicrobial prophylaxis practice patterns: a multi-center, retrospective cohort study

**DOI:** 10.1186/s13756-019-0503-9

**Published:** 2019-03-06

**Authors:** Westyn Branch-Elliman, Steven D. Pizer, Elise A. Dasinger, Howard S. Gold, Hassen Abdulkerim, Amy K. Rosen, Martin P. Charns, Mary T. Hawn, Kamal M. F. Itani, Hillary J. Mull

**Affiliations:** 1 Department of Medicine, Section of Infectious Diseases, VA Boston Healthcare System, MA 1400 VFW Parkway West Roxbury, Boston, MA 02132 USA; 2 Center for Healthcare Organization and Implementation Research (CHOIR), VA Boston, Healthcare System, 150 South Huntington Avenue, Boston, MA 02130 USA; 3 Harvard Medical School, 25 Shattuck Street Boston, Boston, MA 02115 USA; 4 Partnered Evidence-based Policy Resource Center (PEPReC), Department of Veterans Affairs, 150 South Huntington Avenue Boston, Boston, MA 02130 USA; 5 Department of Health Law, Policy and Management, Boston University School of Public Health, 715 Albany Street, Boston, MA 02118 USA; 6 VA Quality Scholars Program, Birmingham VA Medical Center, Birmingham, 700 19th Street S, AL 35233 England; 7 Beth Israel Deaconess Medical Center, Division of Infectious Diseases, 110 Francis Street, Boston, MA 02115 USA; 8 Department of Surgery, Boston University School of Medicine, 88 East Newton Street, C515, Boston, MA 02118 USA; 9 Palo Alto VA Medical Center, 3801 Miranda Ave, Palo Alto, CA 95010 USA; 10 Stanford University School of Medicine, 291 Campus Drive Stanford, Stanford, CA 94305 USA; 11 Department of Surgery, VA Boston Healthcare System, 1400 VFW Parkway West Roxbury, Boston, MA 02132 USA

**Keywords:** Ambulatory surgery, Antimicrobial stewardship, Quality improvement, Prophylaxis, Surgical quality

## Abstract

**Background:**

Guidelines recommend discontinuation of antimicrobial prophylaxis within 24 h after incision closure in uninfected patients. However, how facility and surgical specialty factors affect the implementation of these evidence-based surgical prophylaxis guidelines in outpatient surgery is unknown. Thus, we sought to measure how facility complexity, including ambulatory surgical center (ASC) status and availability of ancillary services, impact adherence to guidelines for timely discontinuation of antimicrobial prophylaxis after outpatient surgery. A secondary aim was to measure the association between surgical specialty and guideline compliance.

**Methods:**

A multi-center, national Veterans Health Administration retrospective cohort from 10/1/2015–9/30/2017 including any Veteran undergoing an outpatient surgical procedure in any of five specialties (general surgery, urology, ophthalmology, ENT, orthopedics) was created. The primary outcome was the association between facility complexity and proportion of surgeries not compliant with discontinuation of antimicrobials within 24 h of incision closure. Data were analyzed using logistic regression with adjustments for patient and procedural factors.

**Results:**

Among 153,097 outpatient surgeries, 7712 (5.0%) received antimicrobial prophylaxis lasting > 24 h after surgery; rates ranged from 0.4% (eye surgeries) to 13.7% (genitourinary surgeries). Cystoscopies and cystoureteroscopy with lithotripsy procedures had the highest rates (16 and 20%), while hernia repair, cataract surgeries, and laparoscopic cholecystectomies had the lowest (0.2–0.3%). In an adjusted logistic regression model, lower complexity ASC and hospital outpatient departments had higher odds of prolonged antimicrobial prophylaxis compared to complex hospitals (OR ASC, 1.3, 95% CI: 1.2–1.5). Patient factors associated with higher odds of noncompliance with antimicrobial discontinuation included younger age, female sex, and white race. Genitourinary and ear/nose/throat surgeries were associated with the highest odds of prolonged antimicrobial prophylaxis.

**Conclusions:**

Facility complexity appears to play a role in adherence to surgical infection prevention guidelines. Lower complexity facilities with limited infection prevention and antimicrobial stewardship resources may be important targets for quality improvement. Such interventions may be especially useful for genitourinary and ear/nose/throat surgical subspecialties. Increasing pharmacy, antimicrobial stewardship and/or infection prevention resources to promote more evidence-based care may support surgical providers in lower complexity ambulatory surgery centers and hospital outpatient departments in their efforts to improve this facet of patient safety.

**Electronic supplementary material:**

The online version of this article (10.1186/s13756-019-0503-9) contains supplementary material, which is available to authorized users.

## Background

Pre-incisional antimicrobial prophylaxis is an effective and standard method for reducing surgical site infections (SSI) prior to high risk clean or clean/contaminated surgical procedures [1]. Continuing antimicrobials after skin closure, however, is not an effective practice for additional SSI reduction [2]. Furthermore, the unnecessary antimicrobial exposure leads to increases in post-operative adverse events, ultimately leading to increased morbidity and excess medical costs [3].

The national Surgical Care Improvement Project (SCIP) included discontinuation of post-operative antimicrobial prophylaxis within 24 h after skin closure following selected inpatient surgical procedures as a core measure, SCIP INF-3 [4]. Up to 48 h was allowed following cardiac surgeries [5]. Prior to implementation of the SCIP INF-3 metric, prolonged courses of antimicrobials were common [6, 7]. However, five years after implementation of this national performance metric, compliance among surgeries reviewed for SCIP exceeded 97% [6]. Due to the marked success of the initiative and high rates of compliance, the antimicrobial discontinuation metric was retired for cases after January 2015 [8].

INF-3 was not included in outpatient SCIP measures and some surgical sub-specialties, including urology, were not included under the umbrella of the discontinuation metric element of the larger program. Thus, compliance with these evidence-based guidelines in outpatient surgical settings is not well characterized. However, recent studies suggest that compliance with evidence-based practice—and early discontinuation of antimicrobials—may be low among many primarily outpatient surgical sub-specialties, including ear/nose/throat (ENT) and urology [9–12]. Prior work suggests that post-operative antimicrobial prophylaxis lasting for greater than 24 h continues to occur in outpatient surgical settings for a variety of reasons, such as the disproven theory that antimicrobials reduce pain and bleeding following tonsillectomies [13]. Other studies suggest that, in some clinical care areas, prolonged antimicrobial prophylaxis is driven by the perception that it is the accepted “standard of care” [14].

Due to the narrow implementation of the SCIP INF-3 measure, dissemination of antimicrobial prophylaxis practices may vary by facility type and by surgical sub-specialty; outpatient surgeries and hospital outpatient departments with inpatient services that were specifically evaluated as part of SCIP may be more likely to adhere than ambulatory surgical centers and surgical specialties that were not included in the program. Another significant factor impacting practice patterns may be the type of ancillary services available at a facility; facilities providing more advanced surgical care may have more ancillary and support services available to assist with development, dissemination, implementation and compliance with evidence-based SSI prevention guidelines.

The Veterans Health Administration (VA) provides surgical care in ambulatory surgery centers (ASCs) and hospital outpatient departments (HOPDs). However, dedicated additional staffing, such as infection preventionists and clinical pharmacy specialists, are only consistently available in facilities with high surgical complexity; low-complexity HOPDs and ASCs have limited access to these services. We hypothesize that the presence of ancillary services, including infectious diseases, infection prevention, antimicrobial stewardship, and advanced pharmacy support in HOPDs leads to lower rates of post-operative prophylactic antimicrobial use and more guideline-concordant care when compared to ASCs where these services are not readily accessible. A secondary aim of our study was to evaluate the association between surgical subspecialty and the rate of guideline-concordant care.

## Methods

### Sample

Our sample included outpatient surgeries performed between Oct. 1, 2015 through Sept. 30, 2017 in VA facilities with surgical programs (111 HOPDs, 26 ASCs). Outpatient surgeries were identified from the outpatient encounter files in the VA Corporate Data Warehouse (CDW) [15]. We included Current Procedural Terminology (CPT) codes with a minor or major outpatient surgery based on the 2015 Surgical Software Classifications developed by the Healthcare Cost and Utilization Project (HCUP) [16].

### Exclusions

Inpatient-only CPT codes, based on Centers for Medicare and Medicaid Service (CMS) guidelines and the VA CPT matrix, were excluded as the focus of the study was outpatient surgeries. The sample was also limited to the most common types of surgical procedures performed, in order to ensure a sufficient volume of HOPD and ASC surgeries. Facilities with fewer than 10 procedures by surgery type during the study period and/or without any procedures in ASCs were excluded. Outpatient surgeries performed by the small subset of providers who performed surgeries in both HOPDs and nearby ASCs were excluded in order to isolate the effects of the facility as opposed to the provider. Lastly, because some patients undergo outpatient surgery as part of the management of an existing infection, skin/soft tissue surgeries and surgeries where the patient had an antimicrobial with > 1 days supplied and filled between 1 and 14 days prior to their procedure date were not included in the cohort.

### Post-operative prophylactic antimicrobial exposure

Post-operative prophylactic antimicrobial exposure was defined as a fill for an antimicrobial within 24 h of the outpatient procedure by date and time stamp recorded in the VA electronic medical record (CDW files). Guideline-concordant care was defined as antimicrobials stopped within 1 day of the procedure; courses lasting longer were defined as prolonged post-procedural prophylaxis. Based on prior research suggesting that prolonged post-operative prophylaxis is typically prescribed for 3–14 days, [10–12] cases with antimicrobial durations lasting for > 14 days were excluded, as were cases who were prescribed antimicrobials > 1 day prior to the procedure. Qualifying systemic antimicrobials were determined in previous work by an infectious disease physician [17]. Topical antimicrobials, including antimicrobial eye drops for ophthalmic procedures, were not measured.

### Covariates

Our objective was to test the association between facility effects and post-operative antimicrobial exposure. The VA defines five levels of facility complexity: ambulatory basic (*n* = 18), ambulatory advanced (*n* = 8), standard inpatient (*n* = 11), intermediate inpatient (*n* = 30) and complex inpatient hospitals (*n* = 70) [18]. We collapsed the two ambulatory facilities and compared the various types of facilities against complex HOPDs (n = 70). The analysis included adjustments for patient characteristics, including patient’s age, race, sex and a weighted comorbidity index based on 12-month prior comorbidities (Elixhauser comorbidities and the Van Walveran composite) [19]. Variation in procedural complexity using the relative value units (RVU) associated with the procedure was also included in the adjusted models. Temporal changes were accounted for using the month and year of the procedure.

Rates of post-operative prophylactic antimicrobial use and patient, procedure and facility characteristics were compared between outpatient surgeries with and without post-operative antimicrobials. Differences between the two groups were assessed using chi-square and t-tests as appropriate. This analysis included evaluating the differences in antimicrobial use rates across surgical sub-specialties. The leading CPT codes associated with post-operative prophylactic antimicrobial use were assessed and then logistic regression models with and without controls for patient, procedure and temporal characteristics were estimated in order to test our hypothesis that facility characteristics were significantly associated with increases in post-operative prophylactic antimicrobial prescribing. Continuous variables (age, comorbidity index and RVU) were converted into to quintiles for more straightforward interpretation in the regression model. All analyses were completed using SAS 9.2. [20]

The local institutional review board approved the study with a waiver of informed consent prior to data collection and analysis.

## Results

**Fig. 1:**
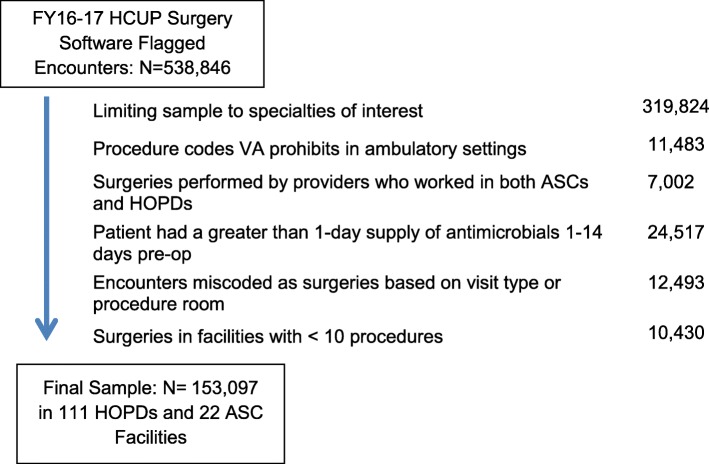
Identification of Study Sample from FY16–17 Outpatient Encounters in VA Facilities with Surgical Services

The final sample included 153,097 outpatient surgeries in 70 complex HOPDs, 30 intermediate HOPDs, 11 standard HOPDs and 22 ASCs after exclusions (See Fig. 1 for Cohort Creation Diagram). Among the five surgical specialties in our final sample (general surgery, which included gastrointestinal procedures such as cholecystectomies, eye, genitourinary, orthopedic and ENT), the most frequent was eye surgery (25%).

There were 7712 outpatient surgeries with prolonged systemic prophylactic antimicrobials (5.0%). Rates ranged from a maximum of 13.7% following genitourinary surgeries to a minimum of 0.4% for eye surgeries. Table 1 shows the rates of post-operative systemic antimicrobial use across surgical specialties in HOPDs and ASCs. These rates were relatively consistent between facility types; however, ENT had significantly higher rates of prolonged use when surgeries were completed in HOPDs (HOPD rate 9.2% versus ASC rate 6.3%, *p* = 0.01) General surgery had the opposite relationship, with post-operative antimicrobial rates of 3% following procedures performed in ASCs and 1% following procedures performed in HOPDs, *p* < 0.001.

**Table 1:** Comparison of Rates of Prolonged Prophylactic Antimicrobial Use across Surgical Specialties in VA ASCs vs. HOPDs

Surgery Type	Total	*n* (%) with Antimicrobials	HOPD		ASC	
			Total	*n* (%) with Antimicrobials	Total	*n* (%) with Prolonged Post-Operative Antimicrobials
Eye	67,778 (25.1%)	254 (0.4%)	64,034	243 (0.38%)	3744	11 (0.29%)
Genitourinary	38,507 (14.3%)	5288 (13.7%)	37,023	5061 (13.67%)	1484	227 (15.30%)
Orthopedics	17,221 (6.4%)	667 (3.9%)	16,712	651 (3.90%)	509	16 (3.14%)
Ear/Nose/Throat	15,661 (5.8%)	1419 (9.1%)	15,054	1381 (9.17%)	607	38 (6.26%)*
General Surgery	13,930 (5.2%)	84 (0.6%)	13,616	75 (0.55%)	314	9 (2.87%)**

Note: Chi-square tests compared the rates of prolonged post-operative antimicrobial use by specialty in hospital outpatient departments (HOPDs) versus ambulatory surgical centers (ASCs) in VA FY16–17 outpatient surgeries. * = *p* < 0.05, ** = *p* < 0.001

5% of the sample was female and the mean age was 68 years (Table 2). Patients undergoing outpatient surgeries were significantly more likely to have prolonged prophylactic antimicrobials if they were female, white, had a more complex procedure, underwent genitourinary or ENT surgeries, or received care in a standard or intermediate HOPD. General surgery and eye surgeries were associated with significantly lower rates of prolonged prophylactic antimicrobials, as was receipt of care in a complex HOPD. Patient co-morbidity score did not impact the receipt of prolonged prophylactic antimicrobials. The mean days supplied was 5 (standard deviation, 3 days), with peaks at 3 days (27%) 5 days (18%) and 7 days (20%); 85% of patients filled prescriptions for 7 days or less (see Additional file 1: Appendix 1). Rates of prolonged prophylactic antimicrobials rates were relatively stable over time for most surgical specialties but there was significant fluctuation in urology and ENT (Additional file 1: Appendix 2).

**Table 2:** Patient, Procedure and Facility Characteristics among FY16–17 VA Outpatient Surgeries in 5 High-Volume Surgical Specialties with and without Prolonged Prophylactic Antimicrobials

Variable	Total (%)	With Prolonged Antimicrobial Exposure, *n* (%)
Total	153,097	7712 (5.0%)
Patient Characteristics		
Female	7623 (5%)	424 (5%)*
White	121,169 (79%)	6191 (80%)*
Age, years (std. dev)	67.6 (11.7)	65.2 (12.5)**
Comorbidity index, score (std. dev)	2.5 (6.5)	2.5 (6.3)
Procedure Characteristics		
Relative value unit (std. dev)	5.4 (3.9)	5.2 (3.5)**
Eye	67,778 (44%)	254 (3%)**
Genitourinary	38,507 (25%)	5288 (69%)**
Orthopedics	17,221 (11%)	667 (9%)**
Ear/Nose/Throat	15,661 (10%)	1419 (18%)**
General Surgery	13,930 (9%)	84 (1%)**
Facility Characteristics		
ASC	6658 (4%)	301 (4%)*
Standard HOPD	3257 (2%)	218 (3%)**
Intermediate HOPD	24,989 (16%)	1839 (24%)**
Complex HOPD	118,193 (77%)	5354 (69%)**

Note: Comparisons are chi-square tests for bivariates and two-tailed t-tests for continuous variables. Symbols indicate significance: **p-value< 0.001; *p-value< 0.05

The top 25 most commonly performed outpatient procedures within the national VA accounted for 76% of all outpatient surgeries in the sample and 61% of surgeries with post-operative antimicrobial exposure (Table 3). Cystoscopies and cystoureteroscopy with lithotripsy procedures had the highest rates of prolonged prophylactic antimicrobials (16 and 20%), while hernia repair, cataract surgeries, and laparoscopic cholecystectomies had the lowest rates (0.2–0.3%).

**Table 3:** Top 25 Surgical Procedures among 153,097 VA Outpatient Surgeries from FY16–17 with and without Prolonged Prophylactic Antimicrobials*

Surgical Specialty	Procedure and CPT Codes	*N* (% of Total Surgeries)	With Prolonged Antimicrobial Exposure, *n* (%)
Eye	CPT: 67028, injection eye drug	23,987 (16%)	46 (0%)
Eye	CPT: 66984–66,983, cataract surgery w/iol 1 stage	19,995 (13%)	39 (0%)
Genitourinary	CPT: 52214–52,332, cystoscopy and treatment	13,965 (9%)	2283 (16%)
Genitourinary	CPT: 55700–55,705, biopsy of prostate	6583 (4%)	795 (12%)
ENT	CPT: 69220–69,222, clean out mastoid cavity	5128 (3%)	46 (1%)
General Surgery	CPT: 49505–49,507, 49,520–49,525, 49,550–49,553, 49,650–49,651, inguinal hernia repair	4824 (3%)	35 (1%)
Eye	CPT: 66982, cataract surgery complex	4761 (3%)	1 (0%)
Eye	CPT: 66821, after cataract laser surgery	3731 (2%)	5 (0%)
Orthopedic	CPT: 29850–29,886, knee arthroscopy/surgery	3219 (2%)	135 (4%)
Genitourinary	CPT: 52353, 52,356, cystoureteroscopy w/ lithotripsy	2357 (2%)	472 (20%)
Genitourinary	CPT: 52204, cystoscopy w/biopsy(s)	2261 (1%)	369 (16%)
General Surgery	CPT: 47562–47,564, laparoscopic cholecystectomy	2208 (1%)	6 (0%)
Orthopedic	CPT: 20680–20,670, removal of support implant	1904 (1%)	146 (8%)
Orthopedic	CPT: 29806–29,825, shoulder arthroscopy/surgery	1888 (1%)	27 (1%)
Genitourinary	CPT: 51102, drain bl w/cath insertion	1792 (1%)	43 (2%)
Eye	CPT: 65855, trabeculoplasty laser surgery	1745 (1%)	2 (0%)
General Surgery	CPT: 49561–49,566, 49,652–49,656, ventral hernia repair	1634 (1%)	4 (0%)
Eye	CPT: 15823–15,822, 67,880–67,882, 67,961–67,966, revision of eyelid, upper or lower	1628 (1%)	64 (4%)
ENT	CPT: 31510, 31,535–31,536, 31,576, laryngoscopy w/ biopsy	1592 (1%)	45 (3%)
Eye	CPT: 66761–66,762, revision of iris	1493 (1%)	4 (0%)
Eye	CPT: 67800–67,808, 67,840, remove eyelid lesion(s)	1449 (1%)	9 (1%)
General Surgery	CPT: 49585–49,586, umbilical hernia repair	1401 (1%)	3 (0%)
ENT	CPT: 38500–38,525, biopsy/removal lymph nodes	1289 (1%)	27 (2%)
Genitourinary	CPT: 52601, prostatectomy (TURP)	1283 (1%)	62 (5%)
Orthopedic	CPT: 20220–20,225, bone biopsy trocar/needle	1003 (1%)	12 (1%)

Note, top 25 surgeries represent 74% of total surgeries and 61% of surgeries with prolonged prophylactic antimicrobials in sample

In the unadjusted logistic regression model predicting prolonged prophylactic antimicrobial use by facility complexity, procedures performed in standard and intermediate HOPDs had significantly higher odds of prolonged prophylactic antimicrobials compared to procedures performed in complex HOPDs (Table 4). The c-statistic in the unadjusted model was 0.544. After controlling for patient, procedure and temporal characteristics, facility type where the procedure was performed remained significantly associated with administration of prolonged systemic prophylactic antimicrobials for both lower complexity HOPDs and ASCs. Younger age, female sex, white race and procedure complexity were also significantly associated with receipt of prolonged prophylactic antimicrobials (c-statistic adjusted model, 0.84).

**Table 4:** Patient, Procedure and Facility Characteristics Associated with Receiving Prolonged Prophylactic Antimicrobials among 153,097 VA Outpatient Surgeries from FY16–17

Variables	Unadjusted Model OR (95% CI)	Adjusted Model OR (95% CI)
Facility Characteristics		
Complex HOPD	Ref	Ref
ASC	1 (0.89–1.12)	1.32 (1.16–1.49)
Standard HOPD	1.51 (1.31–1.74)	1.34 (1.16–1.55)
Intermediate HOPD	1.67 (1.59–1.77)	1.74 (1.64–1.85)
Patient Characteristics		
Age: 20–60 years		1.17 (1.07–1.27)
61–67 years		1.01 (0.93–1.09)
68–70 years		1.09 (1.01–1.19)
71–76 years		1.03 (0.94–1.12)
77–100 years		Ref
Female		1.2 (1.08–1.34)
White		1.1 (1.04–1.17)
Procedure Characteristics		
Relative value unit: 0.5–1.4 RVUs		Ref
1.5–2.8 RVUs		4.03 (3.47–4.68)
2.8–6.4 RVUs		5.83 (5.04–6.75)
6.5–8.5 RVUs		6.27 (5.38–7.31)
8.5–23.5 RVUs		4.49 (3.83–5.26)
Surgical specialty: Orthopedics		Ref
Eye		0.15 (0.12–0.17)
Genitourinary		4.94 (4.51–5.41)
Ear/Nose/Throat		4.19 (3.79–4.63)
General Surgery		0.16 (0.13–0.2)
Model Performance		
C-statistic	0.544	0.836
Max likelihood estimate intercept (std. dev)	−3.05 (0.01)***	−5.31 (0.1)***

Note: Insignificant variables (comorbidity score) and temporal effects not reported

***p-value< 0.0001

## Discussion

Multi-society guidelines recommend against prolonged post-operative antimicrobial surgical prophylaxis, and the most recent comprehensive guidelines for the prevention of surgical site infections make a strong recommendation against any antibiotics following skin closure in the absence of acute infection [1, 21]. However, despite long-standing and high quality evidence that the practice is ineffective and that each additional day of antimicrobial prophylaxis increases preventable harm [22], prolonged post-operative prescribing was common until the introduction of the SCIP measure that specifically discouraged the practice [6]. Among inpatient surgical procedures covered under the SCIP timely discontinuation metric (SCIP INF-3), compliance improved over time and ultimately exceeded 97%. Due to the marked success of the program, SCIP INF-3 was retired as of January 1, 2015 [8]. Notably, the scope of this highly effective metric was limited only to a specific subset of major inpatient surgical procedures; outpatient procedures and some surgical subspecialties, including ENT and urology, were not included. In this large, multi-center, national VA cohort, we determined that implementation of early discontinuation of post-operative antimicrobial prophylaxis was strongly influenced by both surgical subspecialty and facility type; procedures performed within a large, complex hospital system were less likely to receive inappropriate prolonged post-procedural antimicrobial prophylaxis than the same procedures performed in ASCs and low-complexity hospital settings; this is supported by other recent work demonstrating high rates of inappropriate prolonged post-operative antimicrobial use in other outpatient and community settings [11, 12]. There was also a strong influence of surgical subspecialty type on the practice; prolonged prophylaxis was uncommon following orthopedic and general surgeries, which had procedures covered under the inpatient SCIP INF-3, but was frequent following ENT and urology procedures—which did not. High rates of antimicrobial use found among urologic procedures were despite the 2008 American Urologic Association guideline, which recommends less than or equal to 24 h of antimicrobial prophylaxis for all procedures. Additionally, the 2007 American Heart Association guideline recommends against antimicrobial prophylaxis for genitourinary procedures solely to prevent endocarditis in patients with prosthetic valves, further underscoring the scope of the over-use in this specialty [23, 24].

Our data suggest sustained practice change among the surgical subspecialties for which timely discontinuation of surgical antimicrobial prophylaxis was promoted by efforts to comply with SCIP INF-3. These findings are encouraging as they suggest that once the practice is ingrained and integrated into clinical care—and regarded as the “standard of care” by all clinicians in the specialty— the need for ongoing oversight, measurement and reporting may not be necessary. In addition, among INF-3 covered specialties, practice changes impacted not only the specific procedures reviewed for SCIP, but the positive practice changes may have disseminated throughout the surgical subspecialty and improved care— including among surgical procedures that were not evaluated as part of the SCIP program. For example, orthopedic total joint replacement procedures were covered, but less invasive arthroscopic procedures were not evaluated. Among these less invasive procedures, post-operative antimicrobial use is at a very low level, particularly in complex hospital settings.

However, despite these encouraging findings about the success of the INF-3 measure, subspecialty practice islands remain: ENT procedures were not required to report rates of prolonged post-procedural antimicrobials for SCIP, and rates of inappropriate and guideline-discordant use in this specialty remain high. Critically, although some urology procedures were included in inpatient SCIP, no urology procedures were included in the early discontinuation metric, potentially explaining the ongoing high rate of inappropriate antimicrobial use in this specialty.

Our findings are supported by several recent studies exploring current antimicrobial use practices following minor surgeries and procedures. A recent study used a cross-sectional questionnaire to measure current prolonged prophylaxis practices in outpatient foot surgeries and found that 75% of providers administer systemic oral post-procedure prophylactic antimicrobials, most commonly cephalexin for five to seven days [11]. Recent work on outpatient urology found that rates of prolonged prophylaxis are approximately 22% among community-based urologists – very similar to what we found in this VA population [12]. Other studies in different types of uncovered clinical settings, such as cardiac procedural suites, have found similar trends in inappropriate antimicrobial usage and prescribing; in these areas, clinicians report the practice is primarily driven by the perception that prolonged antimicrobial use is the standard of care rather than a perception that the practice is effective [10, 14]. The proven lack of benefit must be weighed against the evidence that prolonged post-procedural antimicrobial use leads to increases in *Clostridium difficile* infections and acute kidney injuries in a duration-dependent manner; the risk of antimicrobial-associated adverse events increases with each additional day of therapy, highlighting the importance of finding ways to limit this ineffective and harmful practice [22, 25].

Ours is not the first analysis to demonstrate racial disparities in antimicrobial prescribing. Prior work characterizing the practice of prolonged post-procedural antibiotic use following cardiac device procedures within the VA found that patients of Hispanic ethnicity were less likely to receive antibiotics than non-Hispanic white patients [10]. An earlier study examining the effect of race on antibiotic prescriptions for children presenting with respiratory viral illness similarly found a strong association between white race and receipt of antimicrobials [26]. Notably, similar to prolonged post-operative prophylaxis, the prescription of antimicrobials for respiratory viral illnesses is specifically recommended against in the combined American College of Physicians and Centers for Disease Control and Prevention Guidelines and thus represents worse, not better, clinical care [27]. However, because patients and providers may perceive receipt of antimicrobials to be a marker of higher quality of care, it is an important health disparity that requires additional investigation and action.

There are many options to improve guideline compliance. Beyond the impact of policies promoted as part of SCIP, interventions and services available in more complex HOPDs may lead to reductions in unnecessary antimicrobial exposure. These facilities perform complex inpatient surgery and the positive impact of infection prevention and antimicrobial stewardship services may spread to outpatient surgeries and specialties not targeted by large, national quality programs such as SCIP. Thus, identifying ways to make these services available in lower complexity facilities may lead to quality improvements over time. Infection prevention resources could be made available in lower complexity and entirely ambulatory centers through educational, telemedicine and policy sharing initiatives. Underscoring the potential for a telehealth solution, a promising recent VA pilot study demonstrated the feasibility and effectiveness of a telemedicine-based antimicrobial stewardship program for expanding these programs to rural health centers that have minimal ancillary support services [28]. In addition, measures to include existing staff in these facilities, such as incorporating local clinical pharmacists for antimicrobial stewardship interventions, may be another important approach to improve care without requiring additional hiring, which may present logistical and financial challenges for some centers, particularly those serving rural areas.

Our study has several limitations. First, it is possible that some of the post-operative antimicrobial use identified in this study was prescribed for the treatment of infections, rather than for prophylaxis. However, our findings are consistent with other small studies examining rates of post-operative antimicrobial use. The very low rates of prescribing for some surgical subspecialties, such as orthopedics and ophthalmology, suggest that background rates of treatment for infections during the immediate post-operative period are low and that findings are driven primarily by antimicrobal prescriptions given for prophylaxis. We were not able to specifically control for wound class; thus, patients with contaminated wounds were not specifically identified. However, most patients with actively infected wounds at the time of surgery receive antimicrobials prior to the procedure and patients with antimicrobials initiated during the 1–14-day window before surgery were specifically excluded, thus mitigating the concern that antimicrobials were prescribed as part of the treatment for dirty or infected cases. Data are from a predominantly male VA population, and thus findings might be different in other patient populations. Our analysis did find that women were more likely to receive prolonged post-operative prophylaxis and it is possible that in populations with a higher proportion of female patients, the practice is more, rather than less, pronounced than was quantified within the VA.

There are also several strengths of our research. The clinical data extracted from the VA electronic medical record allowed us to control for a variety of patient and procedure factors as well as isolate a sample of cases we are confident were not experiencing an ongoing infection. Unlike the private sector, VA surgical facilities are carefully monitored to ensure compliance with infrastructure requirements for complexity ratings; therefore, we were able to model the relationship between these complexity levels and antimicrobial use with more control over facility variation. This is reflected in the high c-statistic in our adjusted models. Lastly, a strength of the analysis was the inclusion of a very diverse collection of types and locations of surgical facilities.

## Conclusions

Among patients undergoing outpatient surgical procedures, factors associated with prolonged antimicrobial prophylaxis include facility complexity and surgical subspecialty. Services that support evidence-based practices, such as policy interventions, pharmacy interventions and antimicrobial stewardship support that are available in more complex facilities may lead to increased dissemination and implementation of and compliance with guideline-driven care. Such interventions may be especially useful for genitourinary and ear/nose/throat surgical subspecialties. Expanding policies and support services to lower complexity facilities with fewer resources, such as through an integrated telemedicine antimicrobial stewardship program, may lead to patient safety improvements.

## Additional file

Additional file 1: Appendix 1. Days Supply for Medication among 7712 VA Outpatient Surgeries from FY16–17 Receiving Prolonged Prophylactic Antimicrobials. Appendix 2. Rates of Prolonged Prophylactic Antimicrobials by Surgical Specialty among 153,097 VA Outpatient Surgeries from FY16–17. (DOCX 83 kb)

## Abbreviations

ASC: Ambulatory surgical center
CDW: Corporate Data Warehouse
CMS: Centers for Medicare and Medicaid Services
CPT: Current procedural terminology
ENT: Ear, nose, and throat
FY: Fiscal year
HCUP: Healthcare Cost and Utilization Project
HOPD: Hospital outpatient department
OR: Odds ratio
RVU: Relative value units
SCIP: Surgical Care Improvement Project
SSI: Surgical site infection
VA: Veterans Affairs
