# Supplementary material for: Facility type and surgical specialty are associated with suboptimal surgical antimicrobial prophylaxis practice patterns: a multi-center, retrospective cohort study
Source: Antimicrob Resist Infect Control. 2019 Mar 6;8:49. doi: 10.1186/s13756-019-0503-9 (PMC6404270; doi:10.1186/s13756-019-0503-9)

**Additional file 1:** Appendix 1. Days Supply for Medication among 7,712 VA Outpatient Surgeries from FY16-17 Receiving Prolonged Prophylactic Antimicrobials


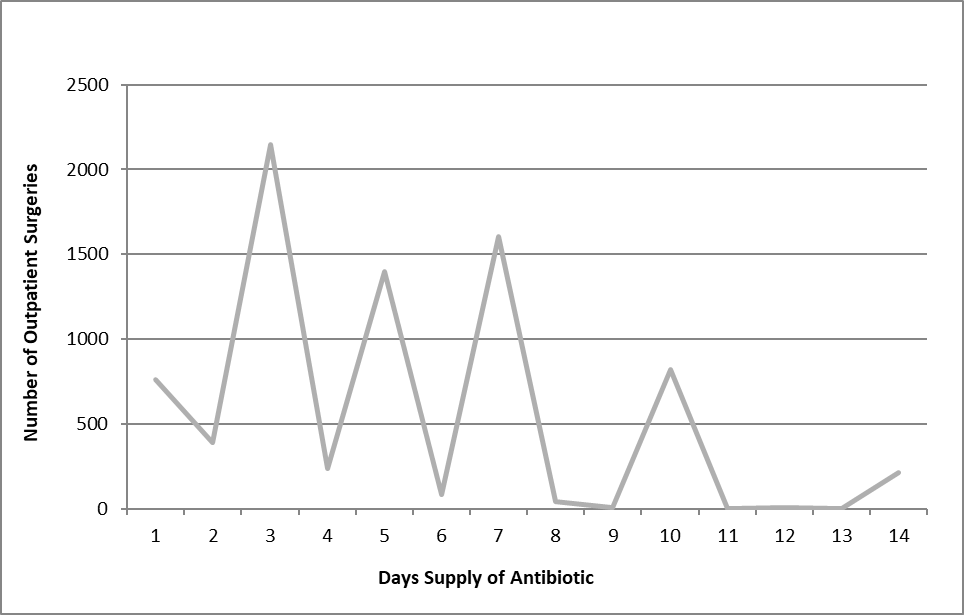


**Additional file 1: Appendix 2**. Rates of Prolonged Prophylactic Antimicrobials by Surgical Specialty among 153,097 VA Outpatient Surgeries from FY16-17


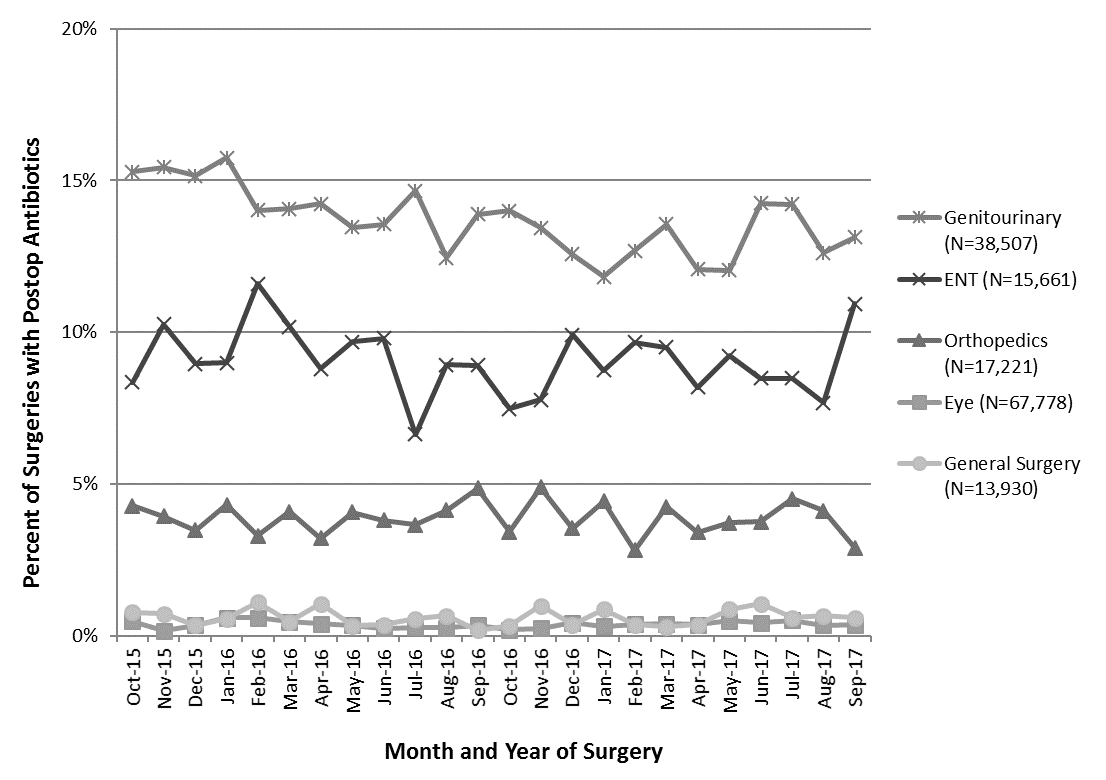

Supplement: Supplementary file 1 — Appendix 1. Days Supply for Medication among 7712 VA Outpatient Surgeries from FY16–17 Receiving Prolonged Prophylactic Antimicrobials. Appendix 2. Rates of Prolonged Prophylactic Antimicrobials by Surgical Specialty among 153,097 VA Outpatient Surgeries from FY16–17. (DOCX 83 kb) [file 13756_2019_503_MOESM1_ESM.docx]
